# Simulation of electrical conductivity for polymer silver nanowires systems

**DOI:** 10.1038/s41598-022-25548-w

**Published:** 2023-01-02

**Authors:** Ali Mohammadpour-Haratbar, Yasser Zare, Kyong Yop Rhee

**Affiliations:** 1 Biomaterials and Tissue Engineering Research Group, Department of Interdisciplinary Technologies, Breast Cancer Research Center, Motamed Cancer Institute, ACECR, Tehran, Iran; 2 Department of Mechanical Engineering (BK21 Four), College of Engineering, Kyung Hee University, Yongin, Republic of Korea

**Keywords:** Engineering, Materials science, Nanoscience and technology

## Abstract

A simple model is developed for the conductivity of polymeric systems including silver nanowires (AgNWs). This model reveals the effects of interphase thickness, tunneling distance, waviness and aspect ratio of nanowires, as well as effective filler volume fraction on the percolation and electrical conductivity of AgNW-reinforced samples. The validity of this model is tested by using the measured data from several samples. Based on this model, the conductivity calculations are in proper accordance with the measured values. A large network and a low percolation onset are produced by nanowires with a high aspect ratio developing the nanocomposite conductivity. The results also show that a thicker interphase expands the network, thereby increasing the electrical conductivity. Furthermore, non-waved AgNWs exhibit more conductivity compared to wavy nanowires. It is concluded that the surface energies of polymer medium and nanowires have no effect on the conductivity of samples. On the other hand, the volume fraction and aspect ratio of nanowires, in addition to the interphase thickness and tunneling distance have the greatest influences on the conductivity of nanocomposites.

## Introduction

Conventional polymer composites contain micrometer-sized particles of organic and inorganic fillers^1^. These materials require high filler content, which can increase the weight of the composite and limit its processing. In consequence, polymers containing high conductive nanofillers such as carbon nanomaterials (such as, carbon nanotubes, graphene and its derivatives) and metallic nanomaterials provide sufficiently conductive polymer nanocomposites (PNCs) at considerably lower nanofiller contents^2,3^. Researchers are exploring PNCs for new applications including biosensors, actuators, energy storage devices such as supercapacitors and batteries, EMI shielding, electrostatic dissipation (EDS), etc^4–6^.

An important property of polymer nanocomposites is their electrical conductivity (EC), which is extremely important for practical applications in electronic devices and sensors^7–9^. Carbon nanotubes and nanowires with high aspect ratios have received special attention as rod-like nanofillers in the fabrication of high conductive PNCs^10,11^. Recent researches have led to the development of metallic nano-materials that have unique electronic, optical, catalytic, magnetic, and thermal properties^12,13^. Owing to the big aspect ratio and superior electrical conductivity, metallic nanowires including copper, gold, nickel, and silver (AgNWs), introduce a major role in the current applications^14,15^. In recent years, AgNWs have attracted much more attention due to their good conductivity and easy to synthesis^16–19^.

AgNWs are one of the most desirable materials since bulk Ag has a high conductivity (6.3 × 10^7^ S/m), which makes them applicable in sensing, electronics, electromagnetic interference (EMI) shielding^20,21^. Furthermore, AgNWs are more human friendly than other non-metallic conductive nanomaterials like carbon nanotubes because they possess antimicrobial properties^22^. Therefore, AgNWs are a promising candidate for the fabrication of conductive polymer/ AgNWs nanocomposites (PAgNWs)^23,24^. An attractive use of AgNW-based PNCs is the manufacture of electrochemical biosensors for the detection of breast cancer^25^. Electrochemical biosensors are a group of sensors that exhibit high sensitivity, fast response, and low manufacturing costs for detecting different types of biological agents and diseases such as diabetes^26,27^, cancer^28^, etc. Since breast cancer is one of the three most dangerous and deadly cancers in women, an early detection can be of great assistance to patients. Consequently, it is necessary to develop the rapid diagnostic devices such as biosensors that possess high sensitivity and selectivity^29^. AgNWs with their high electrical conductivity, antibacterial properties, and high specific surface area are excellent candidates for the manufacture of electrochemical biosensors based on polymer nanocomposites for cancer detection.

At a certain concentration of the conductive nanofiller, commonly known as the electrical percolation threshold, PNC conductivity increases dramatically^30^. At percolation concentration, nanofillers produce conductive network in the polymer matrix^31^. Several parameters affect the percolation threshold and electrical conductivity in PNCs, including aspect ratio and dispersion state of the nanofiller within the polymer matrix, wetting between polymer and nanofiller, interphase thickness, tunneling distance, waviness and agglomeration of nanowires, volume fraction of nanowires, and processing technique^31^.

The key mechanism responsible for PAgNW electrical conductivity is electron tunneling^32^. In other words, the tunneling distance directly impacts the transfer of charges through tunneling regions. Thus, during the electron tunneling process between nanowires, the tunneling size will affect the conductivity of final PNC. Additionally, the interphase regions formed in PNCs due to the rigid interface between polymer medium and nanofiller influence the electrical conductivity. The thickness of these regions, called the interphase thickness, affects the percolation and conductivity of PNCs^33^. As the interphase thickness increases, electrical conductivity increases as well^34^. The calculation of tunneling distance and interphase thickness is challenging in practice and in experiments. So, providing a computational model to estimate these parameters and finally calculate the electrical conductivity of the nanocomposite would be extremely useful and beneficial.

Numerous models have been developed to analyze the conductivity of PNCs. A common method of expressing conductivity is the power-law assuming percolation concept and filler concentration. It is not able to reflect the key features of PNCs such as interphase thickness, waviness, tunneling distance, and nanofiller aspect ratio^35^. Consequently, it is crucial to develop new models for electrical conductivity of PNCs that are capable of taking into account the key factors such as interphase regions, tunneling distance, nanofiller waviness, and aspect ratio.

In our previous article^34^, Kovacs model was developed by considering logical parameters for approximating of electrical conductivity in the AgNW-filled samples. We have investigated the effects of various parameters including volume fraction and dimensions of nanofiller, as well as interphase thickness and tunneling resistivity in the previous model. However, one problem was the estimating of tunneling resistivity in that model. In the present work, Taherian model is developed to estimate the electrical conductivity of AgNW-filled composites. Using this simple and applicable model, it is possible to examine the impacts of curliness, interphase thickness, aspect ratio, the volume fraction of nanowires, and tunneling size on the conductivity of AgNW composites. Using the experimented results from the literature, the proposed model is examined and interphase/tunneling parameters are calculated. More, the stimuli of factors on the conductivity of samples are studied. Authors hope that this model will be able to aid in calculating the electrical conductivity of PNCs. Most previous studies have calculated the electrical conductivity of AgNW nanocomposites using numerical methods. However, the previous models commonly disregarded the interphase depth and tunneling properties. Also, previous model cannot present the conductivity by a simple way. Actually, we present a simple model by meaningful and effective parameters controlling the conductivity of AgNW-filled composites.

## Methods and modeling

For predicting the electrical conductivity of PNCs reinforced with conductive nanofillers, Taherian^36^ developed a mathematical model based on three key factors, including conductivity of filler, aspect ratio of particles, and wettability between medium and nanoparticle as follows:

1 $${\upsigma } = {\upsigma }_{{\text{m}}} + { }\frac{{{\text{P}}\upalpha {\upsigma }_{{\text{f}}} }}{{1 + {\text{Q exp}}\left( { - \frac{{{\text{roundness}}}}{{{\text{cos}\upbeta }}}} \right)}}$$

“σ_m_” and “σ_f_” are the conductivity of the polymer and nanofiller, respectively, “α” displays the. aspect ratio of nanofiller, roundness depends on “α”, cos (β) represents the wettability between polymer and nanofiller, and “P” and “Q” are also changeable parameters. The electrical conductivity of the majority of polymers is very low. Therefore, there is no need to include the negligible level of “σ_m_” in model. Consequently, Eq. (1) is modified as:

2 $${\upsigma } = \frac{{{\text{P}}\upalpha {\upsigma }_{{\text{f}}} }}{{1 + {\text{Q exp}}\left( { - \frac{{{\text{roundness}}}}{{{\text{cos}\upbeta }}}} \right)}}$$

In an article published in 2008, authors offered a model for the conductivity of PNCs containing carbon nanotubes with a random distribution^37^ as:

3 $${\upsigma } = {\upsigma }_{{\text{m}}} + \frac{{{\text{f }}\emptyset_{f } {\upsigma }_{{\text{f}}} }}{3}$$

"$\emptyset_{f }$" refers to the nanofiller volume fraction, and "f" refers to the portion of particles in the network. Therefore, "$\emptyset_{f }$" and "f" directly affect the conductivity. Thus, "P" parameter can be regarded as a function of "$\emptyset_{f }$" and "f" by comparing Eqs. (2 and 3). Here, we consider "$\emptyset_{f }$" and "f" by nonlinear functions and Eq. (2) is rewritten as:

4 $${\upsigma } = \frac{{{\text{f}}^{3} { }\emptyset_{f }^{3} \upalpha {{ \upsigma }}_{{\text{f}}} }}{{1 + {\text{Q exp}}\left( { - \frac{{{\text{roundness}}}}{{{\text{cos}\beta }}}} \right)}}$$

In view of the fact that electrical conductivity is a nonlinear function of filler concentration, considering nonlinear "$\emptyset_{f }$" and "f" will be closer to reality and experimental data.

Typically, rod-like nanofillers have an aspect ratio (ɑ) of:

5 $$\alpha = { }\frac{{\text{l}}}{{\text{d}}}$$

"l" and "d" represent the length and diameter of the nanofiller.

In addition, there is a percolation threshold for nanofillers with random dispersion^38^ as:

6 $$\emptyset_{{\text{p}}} = { }\frac{{\text{V}}}{{{\text{V}}_{{{\text{ex}}}} }}$$

Nanoparticle volume is represented by "V" and "V_ex_" is an excluded volume as the volume around a particle that is not available to nearby particles. "V" and "V_ex_" in PNCs containing tubular fillers with accidental dispersion are expressed^38^ as:

7 $$V = \frac{\pi }{4}d^{2} l + \frac{\pi }{6}d^{3} = \frac{\pi }{2}d^{2} \left( {0.5l + \frac{d}{3}} \right)$$

8 $$V_{ex} = \frac{4}{3}\pi d^{3} [1 + \frac{3}{2}\alpha + \frac{3}{8}\alpha^{2} ]$$

Using an interphase layer, PNCs can form large networks. There is the following alteration in the excluded volume as a result of the interphase part:

9 $$V_{ex} = \frac{4}{3}\pi \left( {d + 2t} \right)[\left( {d + 2t} \right)^{2} + \frac{3}{2}\left( {d + 2t} \right)l + \frac{3}{8}l^{2} ]$$

“t” represents interphase thickness. The interphase zone is created around the nanowires and has a lower conductivity than nanowires. So, it can transfer the electrons properly.

Further, big rod-like nanofillers cause the waviness that reduces their conductivity. Therefore, for the purpose of calculating the conductivity based on the waviness parameter (u), the equivalent length (l_eq_) needs to be defined^34^ as follows:

10 $$l_{eq} = \frac{l}{u}$$

Nanofiller will have no waviness if u = 1, but if u > 1, it will have more waviness. By considering "l_eq_" as the effective length of nanofillers with high waviness (leq = l/u), "V_ex_" is changed to:

11 $$V_{ex} = \frac{4}{3}\pi \left( {d + 2t} \right)[\left( {d + 2t} \right)^{2} + \frac{3}{2}\left( {d + 2t} \right)\left( \frac{l}{u} \right) + \frac{3}{8}\left( \frac{l}{u} \right)^{2} ]$$

Percolation threshold of nanofillers can be expressed as follows by including waviness and interphase thickness in “V_ex_” formulation:

12 $$\emptyset _{p} = \frac{{\frac{\pi }{4}d^{2} l}}{{\frac{4}{3}\pi \left( {d + 2t} \right)}}\left[ {\left( {d + 2t} \right)^{2} + \frac{3}{2}\left( {d + 2t} \right)\left( {\frac{l}{u}} \right) + \frac{3}{8}\left( \frac{l}{u} \right)^{2} } \right]$$

AgNWs and the surrounding interphase constitute the effective volume fraction of the filler as follows:

13 $$\emptyset_{eff} = \emptyset_{f} + \emptyset_{i}$$

∅ _i_ refers to the volume fraction of the interphase areas, which is given as follows:

The volume fraction of interphase is calculated as:

14 $$\emptyset_{i} = \emptyset_{f} \left( {1 + 2\frac{t}{d}} \right)^{2} - \emptyset_{f}$$

By placing the Eq. (14) into Eq. (13), “*∅ *_*eff*_*”* is expressed as:

15 $$\emptyset_{eff} = \emptyset_{f} \left( {1 + 2\frac{t}{d}} \right)^{2}$$

The conductive networks are produced by only a portion of nanofillers once they reach the percolation threshold, while the remaining nanowires are dispersed throughout the medium. The part of percolated nanowires is considered as follows:

16 $$f = \frac{{\emptyset _{f} ^{{1/3}} - \emptyset _{p} ^{{1/3}} }}{{1 - \emptyset _{p} ^{{1/3}} }}$$

The formula for calculating the fraction of networked nanowires is written as follows by considering “*∅ *_*eff*_*”* instead of “*∅ *_*f*_*”* in the above equation:

17 $$f = \frac{{\emptyset_{eef}^{{{\raise0.7ex\hbox{$1$} \!\mathord{\left/ {\vphantom {1 3}}\right.\kern-\nulldelimiterspace} \!\lower0.7ex\hbox{$3$}}}} - \emptyset_{p}^{{{\raise0.7ex\hbox{$1$} \!\mathord{\left/ {\vphantom {1 3}}\right.\kern-\nulldelimiterspace} \!\lower0.7ex\hbox{$3$}}}} }}{{1 - \emptyset_{p}^{{{\raise0.7ex\hbox{$1$} \!\mathord{\left/ {\vphantom {1 3}}\right.\kern-\nulldelimiterspace} \!\lower0.7ex\hbox{$3$}}}} }}$$

Furthermore, the conductivity of wavy filler is also defined^34^ as:

18 $$\sigma_{AgNWs} = \frac{{\sigma_{f} }}{u}$$

Ryvkina et al^39^. offered a mathematical model for PNCs that emphasizes the electron tunneling mechanism as the dominant mechanism in the conduction of PNCs as:

19 $$\upsigma \sim \exp \left( { - {\text A} \uplambda } \right)$$

In this expression, “A” refers to a characteristic tunnel distance, and “${\uplambda }$” refers to the tunneling distance between nanofillers. The tunneling distance determines the distance between neighboring AgNWs and when is less than 10 nm, it can transfer the electrons and cause the conductivity. Our previous work^34^ showed that the electrical conductivity of PNCs containing AgNWs is inversely proportional to the ${\uplambda }$. In this way, "Q" parameter in Eq. (4) may represent the tunneling distance between nearby nanofillers in PNCs. The "Q" parameter is equal to "${ }\frac{{{\uplambda }^{2} }}{{z^{2} }}$ ". Therefore, Eq. (4) is rewritten as follows:

20 $$\sigma = \frac{{f^{3} \emptyset_{f }^{3} \alpha \sigma_{f} }}{{1 + \frac{{\lambda^{2} }}{{z^{2} }} \exp \left( { - \frac{roundness}{{\cos \beta }}} \right)}}$$

where z = 1 nm as a tunneling factor. In other words, "d" in this equation has nm unit.

In addition, the "roundness" factor was suggested by Taherian^36^. In other words, the roundness increases with a decrease in the aspect ratio of nanofiller. The roundness is measured between 0 and 1. For roundness, Zare et al^35^. have provided the following equation:

21 $${\text{roundness}} = \frac{1000 - \alpha }{{1000}}$$

The role of wetting in electrical conductivity by cos (β) has been proposed by Taherian^36^ as follows:

22 $$\cos \beta = \frac{{\gamma_{f} - \gamma_{fp} }}{{\gamma_{p} }}$$

“$\gamma_{f}$, “$\gamma_{p}$”, and “$\gamma_{fp}$” represent the surface energies of nanofiller, polymer, and filler/polymer interphase, respectively, and "β" denotes the wetting angle. Additionally, “$\gamma_{fp}$” can be defined using the surface energies as follows:

23 $$\gamma_{fp} = \gamma_{p} + \gamma_{f} - 2\left( {\gamma_{f} \gamma_{p} } \right)^{0.5}$$

Equation (20) can be rewritten as follows by substituting Eqs. (21) and (22):

24 $$\upsigma = \frac{{{\text{f}}^{3} ~\emptyset _{f}^{3} \alpha ~\upsigma _{{\text{f}}} }}{{1 + \frac{{\lambda ^{2} }}{{{\text{z}}^{2} }}~\exp \left( { - \frac{{\frac{{1000 - \alpha }}{{1000}}}}{{\frac{{\gamma _{{\text{f}}} - \gamma _{{{\text{fp}}}} }}{{\gamma _{{\text{p}}} }}}}} \right)}}$$

**Figure 1:**
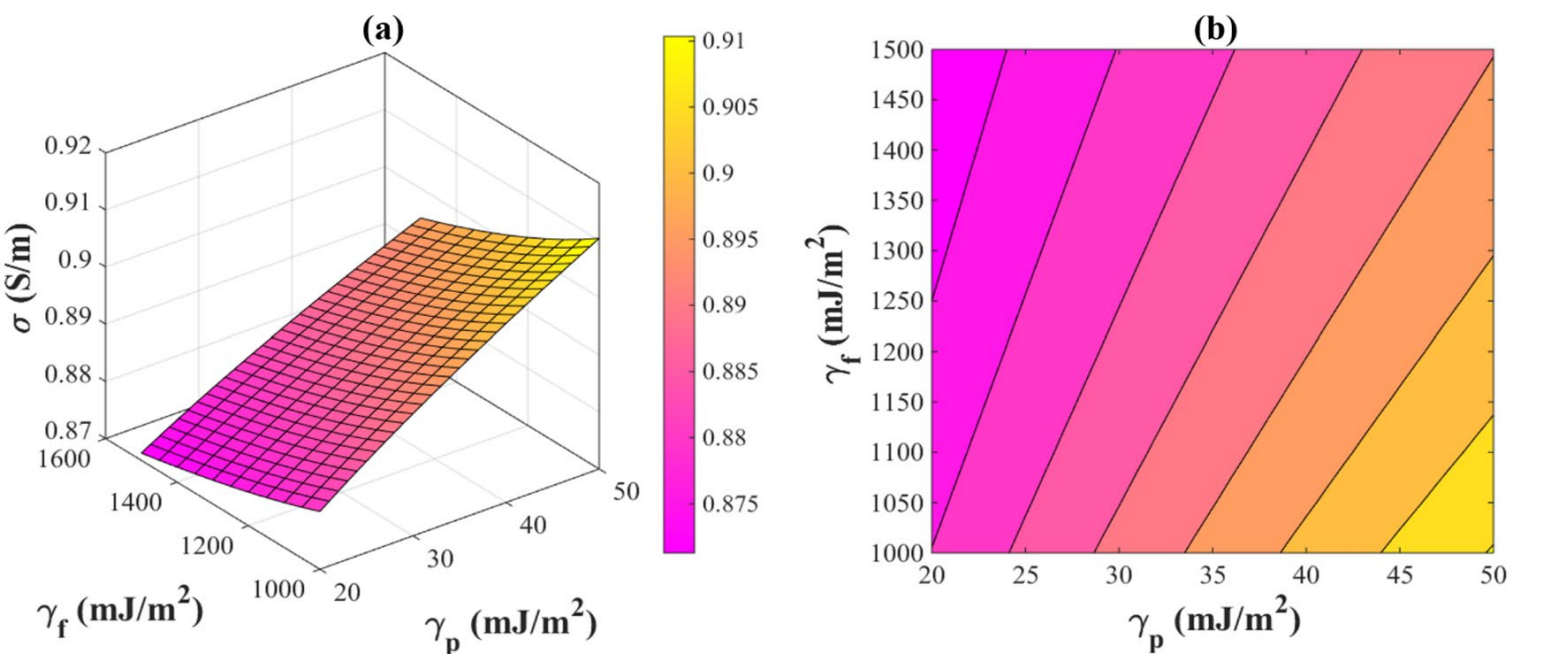
The effects of *“*${\varvec{\gamma}}_{{\varvec{f}}}$ *and “*${\varvec{\gamma}}_{{\varvec{p}}}$*”* on the electrical conductivity of a PNC by (**a**) 3D and (**b**) contour plots.

In Fig. 1, the effects of *“*$\gamma_{f}$ *and “*$\gamma_{p}$*”* on the electrical conductivity of PNCs are illustrated. Polymers have a surface energy of 20–50 mJ/m^2^^40^ and AgNWs have a surface energy of 1000–1500 mJ/m^2^^41^. Figure 1 shows that the electrical conductivity of the PNC insignificantly changes at these intervals. Accordingly, ${\text{exp}}\left( { - \frac{{\frac{1000 - \upalpha }{{1000}}}}{{\frac{{\gamma_{f} - \gamma_{fp} }}{{\gamma_{p} }}}}} \right)$ term in Eq. (24) can be ignored. Hence, Eq. (24) is rewritten as follows:

25 $$\upsigma = \frac{{{\text{f}}^{3} ~\emptyset _{f}^{3} \alpha ~\upsigma _{{\text{f}}} }}{{1 + \frac{{\lambda ^{2} }}{{z^{2} }}~}}$$

Equation 25 offers a simple and beneficial model for estimating of conductivity in the PNCs containing AgNWs. In the present model, the effects of various factors such as aspect ratio, volume portion, and conductivity of nanowires, as well as tunneling length and interphase depth have been reflected.

## Results and discussion

### Proof of model by experiment

**Figure 2:**
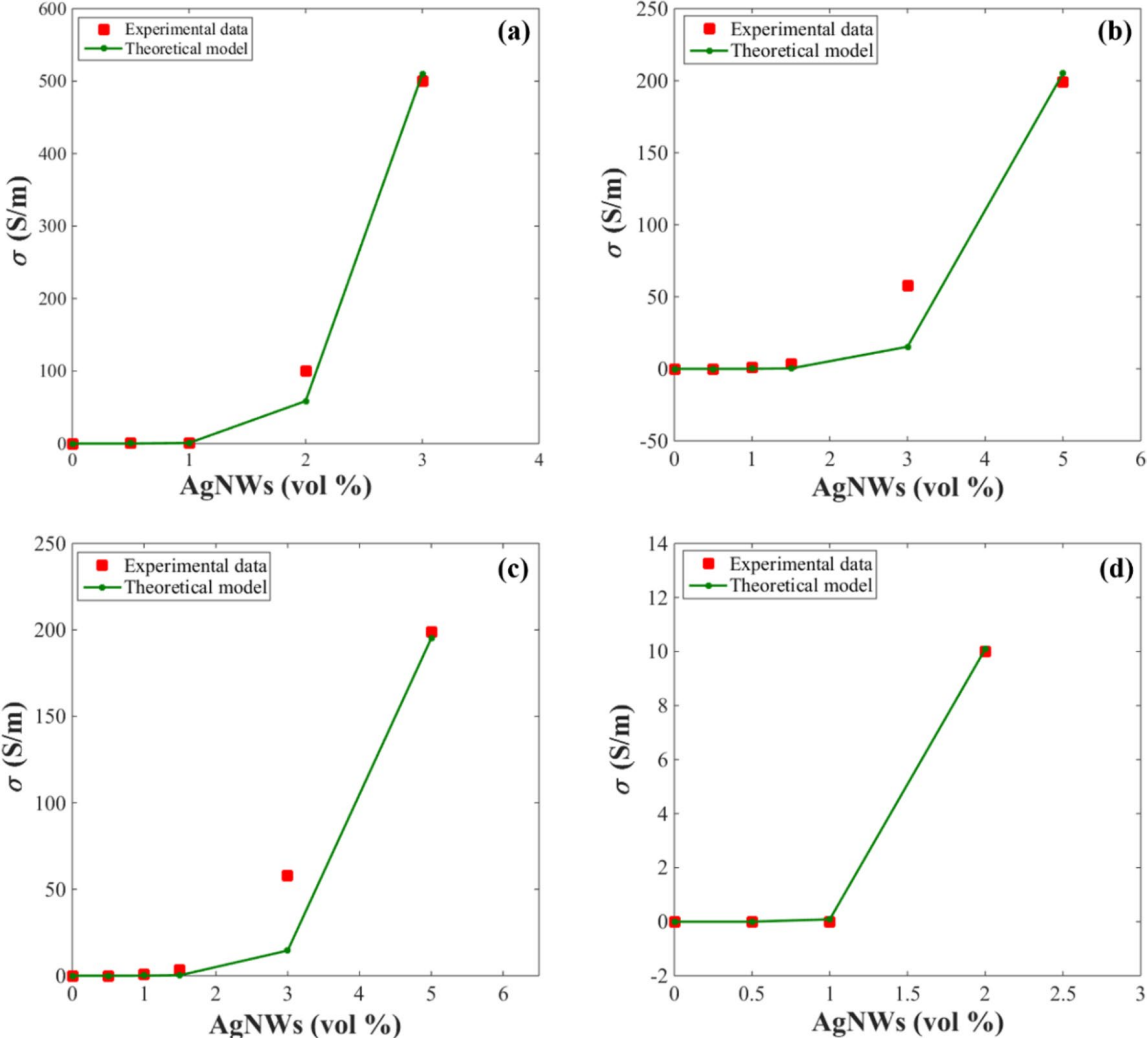
Experimental and theoretical conductivities for (**a**) PI/AgNWs^42^, (**b**) PEKK/AgNWs^43^, (**c**) PLA/AgNWs^23^ , and (**d**) PMMA/AgNWs^44^ samples at different nanofiller concentrations based on the proposed model.

Experimental data from the literature are utilized to prove the rationality of the proposed model. Experimented results and outputs of the offered model are shown in Fig. 2 for polyimide/AgNWs (PI/AgNWs) nanocomposite (*d* = 250 nm, *l* = 50 μm, *u* ≈ 1.45)^42^, poly (ether ketone ketone) (PEKK)/AgNWs sample (*d* = 260 nm, *l* = 55 μm, *u* ≈ 1.65)^43^, poly (lactic acid)/silver nanowire (PLA/AgNWs) nanocomposite (*d* = 250 nm, *l* = 50 μm, *u* ≈ 1.6)^23^, and poly(methyl methacrylate)/silver nanowire sample (PMMA/AgNWs) nanocomposite (*d* = 15 nm, *l* = 2.7 μm, *u* ≈ 1.9)^44^. We obtained the dimensions (d and l) of nanowires for these samples from references^23,42–44^. In Fig. 2, experimented conductivity displays good agreement with the proposed model. As a result, the proposed model is an ideal equation for estimating the conductivity in the real-world applications.

Two key parameters in nanocomposites that affect the electrical conductivity are the interphase thickness and the tunneling distance for electron transfer. Comparing measured data to the forecasts of the proposed model can determine the average values of these parameters. According to the experimental results, $\hbox{``}\emptyset_{p} {^{\prime\prime}}$ was reported as 0.0049 for the PI/AgNWs^42^, 0.0059 for PEKK/AgNWs^43^, 0.0059 for PLA/AgNWs^23^, and 0.008 for PMMA/AgNWs^44^. By applying the experimental values of percolation threshold to Eq. (12), we can calculate the value of “t” and “λ” (t, λ). By comparing the experimental conductivity values with those of the offered model, the values of (t, λ) are found as (5 nm, 1.1 nm), (7 nm, 7.5 nm), (6 nm, 7.5 nm), and (1.5 nm, 4 nm) for the PI/AgNWs^42^, PEKK/AgNWs^43^, PLA/AgNWs^23^, and PMMA/AgNWs^44^ nanocomposites, respectively. Actually, numerous levels for “t” and “λ” are calculated by fitting the measured percolation onset to Eq. (12), but we report the average ones. However, interphase depth should be shorter than the gyration radius of macromolecules and tunneling distance should be lower than 10 nm to encourage the electron transferring. All calculations for interphase depth and tunneling distance fall within the suitable ranges confirming the perditions. Consequently, this model can be used to calculate the interphase thickness and tunneling distance in PNCs. Furthermore, the values of “t” and λ"” obtained by this model are almost identical for two nanocomposites with the same percolation thresholds. Therefore, it is evident that the proposed model is highly accurate.

## Examinations of factors on the conductivity

The offered model is used to investigate how different parameters handle the percolation onset and nanocomposite conductivity. This assessment determines the predictability of the offered model plus the effects of the factors on the electrical conductivity of PNCs. Calculating of electrical conductivity has been done by taking the average of the following parameters: u = 1.4, l = 50 µm, t = 5 nm, d = 200 nm, λ = 2 nm, ∅_f_ = 0.01, and σ_f_ = 6 × 10^7^ S/m.

**Figure 3:**
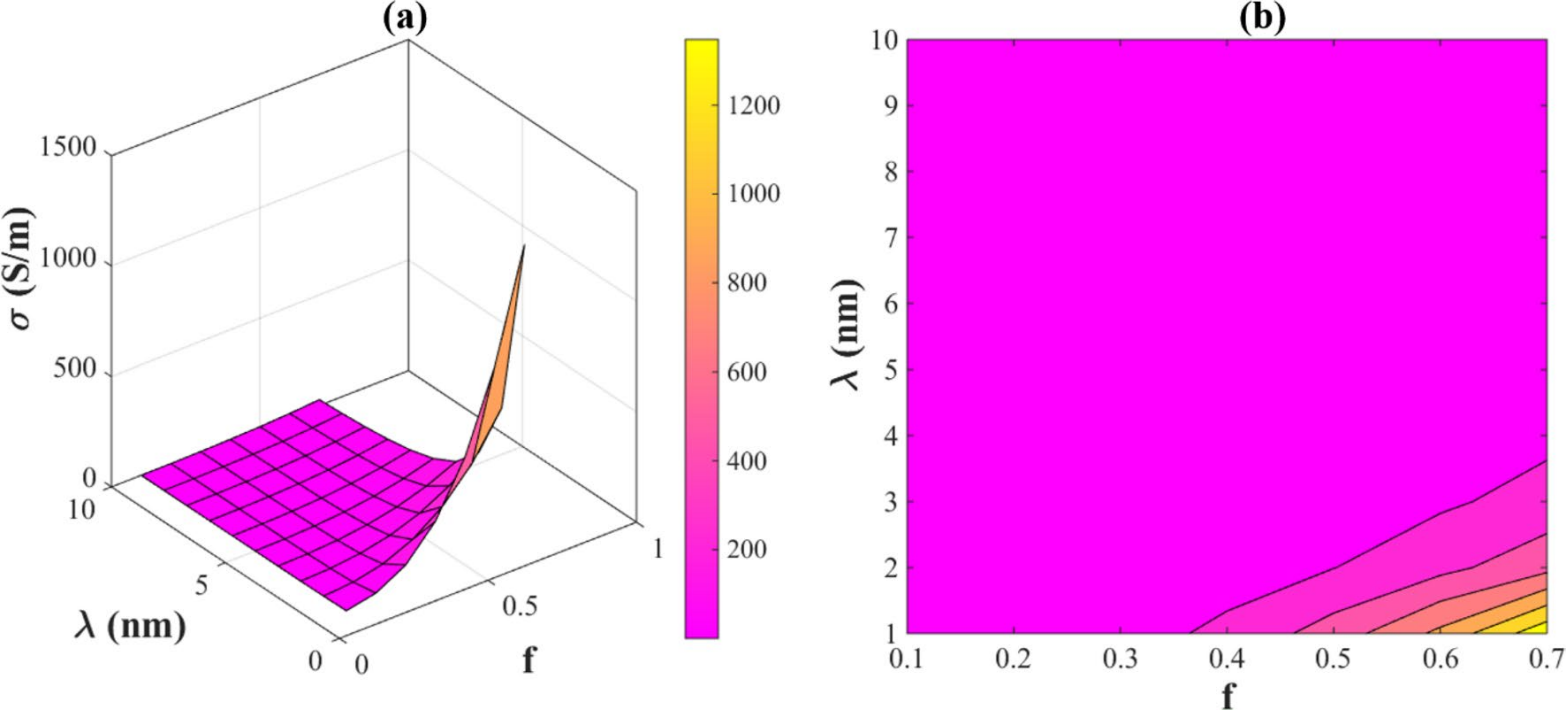
The impacts of *“f” and “λ”* on the electrical conductivity of PNCs by (**a**) 3D and (**b**) contour plots.

Figure 3 illustrates the impresses of “f” and “λ” on the conductivity of PNCs. The conductivity reaches its maximum (1300 S/m) when λ = 1 nm and f = 0.7. We also observe that the conductivity is about 0 at λ > 3.5 nm or f < 0.37. As a result, increases in “f” value in a small tunneling distance between nanowires lead to an improved electrical conductivity of the final PNC. Alternatively, low conductivity is observed when a large number of nanowires cannot participate in the conductive networks and nanowires are far apart. Literature studies have shown that once percolation is achieved, the percentage of networked nanofillers and the tunneling distance between fillers influence the conductivity significantly^45,46^.

Electrical conductivity increases with increasing the network dimensions, whereas electrical conductivity decreases with smaller networks. Therefore, the value of “f” directly correlates with the electrical conductivity. In addition, our previous work^34^ identified a direct influence of network dimension on the conductivity of PNCs, which supports the accuracy of offered model for the calculation of electrical conductivity in PNCs. Additionally, the tunneling effect is a probabilistic phenomenon heavily influenced by the distance between any neighboring nanowire pair^47^. Percolation occurs when electrical links are formed between nanofillers that are physically separated, thereby forming conductive networks^45^. A mechanism of electron tunneling is believed to determine the conductivity. In fact, electrons transfer between nanofillers through electron tunneling^45^. The tunneling distance determines the distance between adjacent AgNWs and can transfer the electrons when it is less than 10 nm. Because of van der Waals interactions, nanofillers cannot physically contact each other. Thus, they remain separated by an energy barrier of a nanometer gap, where electron tunneling may occur when the distance between two nanofillers is less than the threshold cutoff distance^47,48^. There have been several studies indicating a tunneling cutoff distance of about 1.4 nm^49,50^. In addition, numerous reports confirm that electrical conductivity is inversely related to tunneling distance^51–53^. Also, as shown in Eq. (25), conductivity is inversely related to tunneling distance. Therefore, the offered model correctly guesses the effect of tunneling distance on the nanocomposite conductivity.

**Figure 4:**
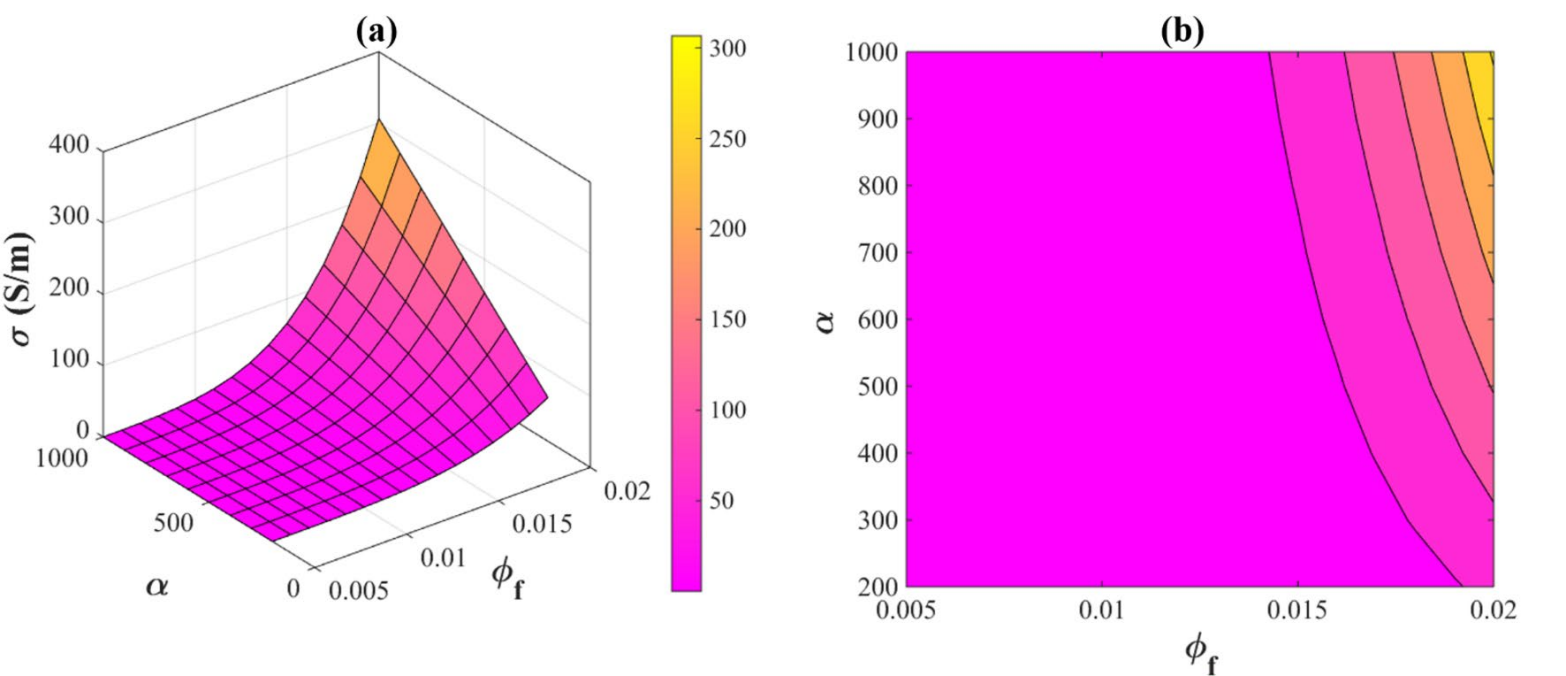
The stimuli of *“∅*_*f*_*” and “α”* on the electrical conductivity of PNCs by (**a**) 3D and (**b**) contour plots.

Figure 4 shows the impressions of “∅_f_” and “α” on the conductivity of PNCs. Electrical conductivity is maximized as 305 S/m at ∅_f_ = 0.02 and α > 820. In contrast, at ∅_f_ < 0.014, the minimum value of conductivity is observed and nanocomposite is insulated. Nanowires with a higher aspect ratio and higher volume fraction will cause an improved electrical conductivity, while a lower aspect ratio and smaller volume fraction of nanowires can result in a reduction in the conductivity.

The nanowires in the polymer matrix begin to form conductive networks after passing the percolation threshold. When nanowire concentrations are below the percolation levels, conductive networks cannot be formed. Therefore, electron tunneling is not possible. In filler concentrations above the percolation threshold, a fraction of nanowires creates the conductive networks with specific dimensions that assist to electron transfer and thus increase the conductivity. Furthermore, increasing the aspect ratio of nanowires improves the electrical conductivity. In other words, the smaller the diameter and the longer the length of the nanowires, the easier the formation of conductive networks in the polymer matrix, thus allowing percolation to take place at lower volume fractions of the nanowires, which improve the electrical conductivity. Moreover, literature studies indicate that higher aspect ratio of conductive particles provides higher electrical conductivity for nanocomposites^54,55^. The level of conductivity above the percolation threshold is equivalent for all kind of particle with same nature of material. A lower percolation threshold shifts the percolation curve to lower amount of fillers and the same level of conductivity is obtained with a lower content of nanowires.

Therefore, the proposed model accurately predicts the relationship between the aspect ratio and the electrical conductivity of nanocomposites.

**Figure 5:**
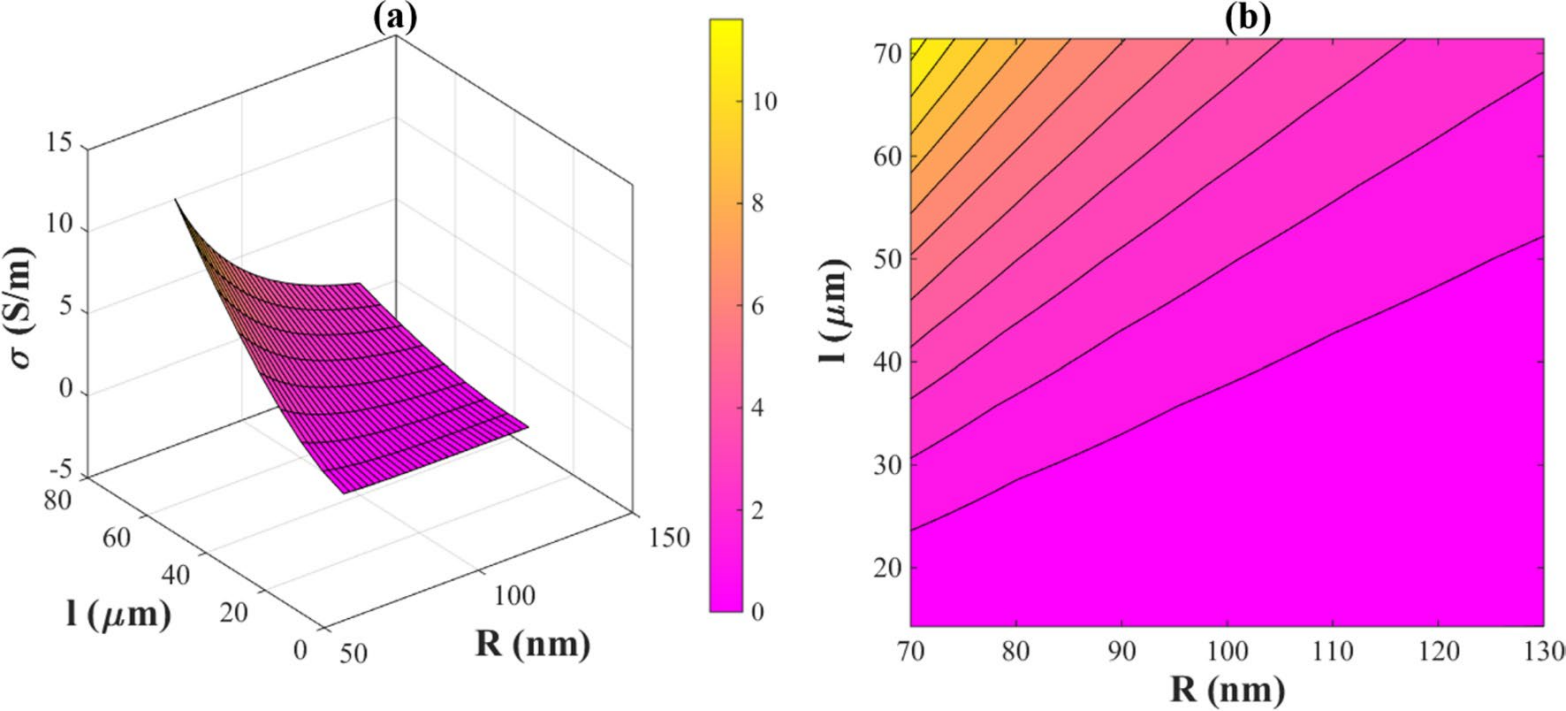
The influences of *“R” and “l”* on the electrical conductivity of PNCs by (**a**) 3D and (**b**) contour plots.

Figure 5 displays the impacts of the radius and length of nanowires on the conductivity. Based on this plot, it is seen that the electrical conductivity is inversely proportional to the radius (or diameter) of the nanowires and directly proportional to their length. A maximum conductivity of 12 S/m is obtained for PNCs with l = 70 μm and R = 70 nm. However, the PNC is a complete insulator at l < 24 μm and all radii of nanowires.

Nanowires with a smaller radius and a bigger length have a greater aspect ratio. At higher aspect ratios of nanowires, conductive networks are easier to form^56^. Consequently, electrical percolation takes place at a lower volume fraction of nanowires. Furthermore, the dimensions of the conductive networks become larger at higher aspect ratios, and electrons can easily tunnel through the nanowires, thus growing the conductivity. On the other hand, nanowires with a larger radius and shorter length cause a lower conductivity for the PNC, because the aspect ratio decreases and conductive networks are difficult to form.

**Figure 6:**
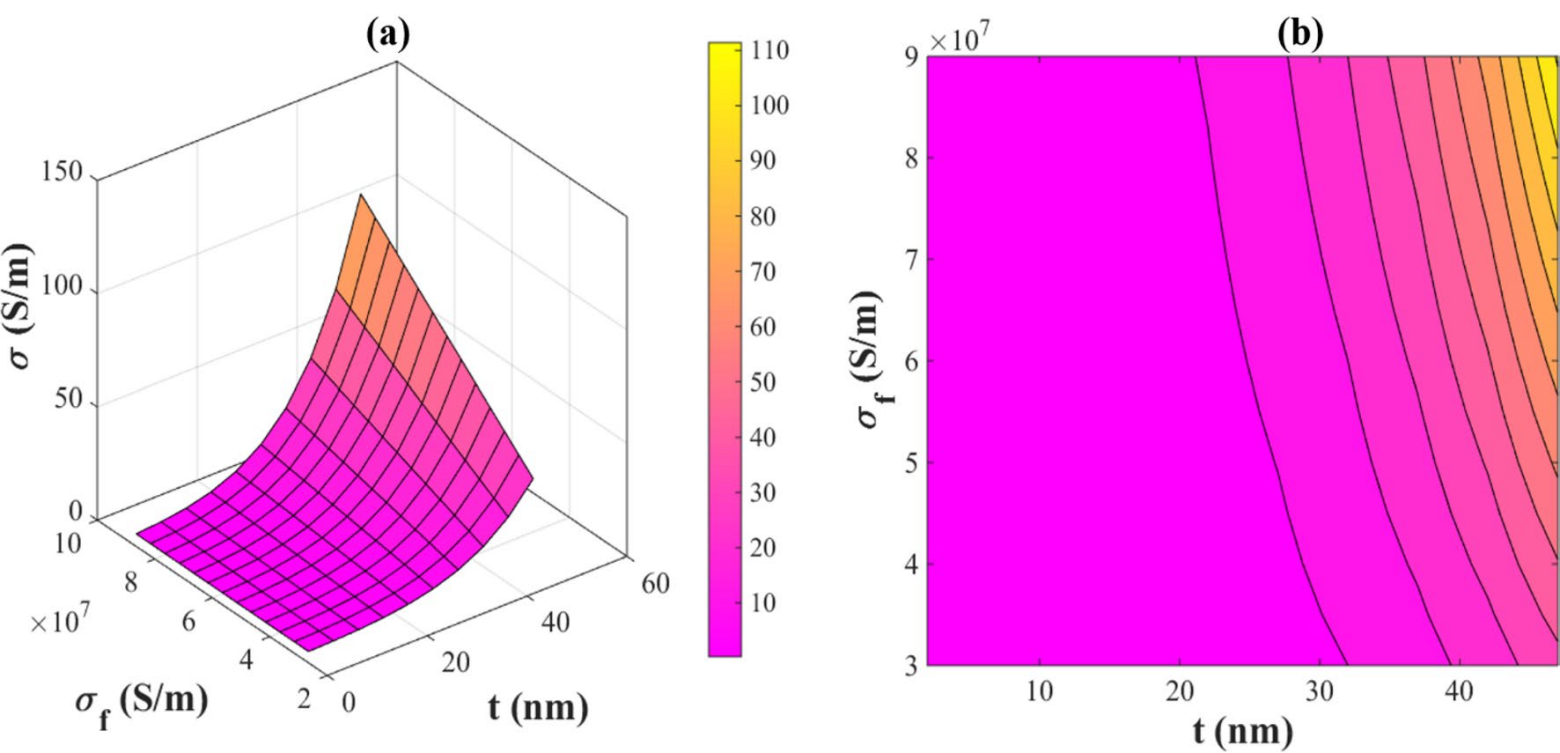
The influences of *“t” and “σ*_*f*_*”* on the electrical conductivity of PNCs by (**a**) 3D and (**b**) contour plots.

The effects of nanowire conductivity and the thickness of interphase on the electrical conductivity of PNCs are also illustrated in Fig. 6. It is illustrated that the electrical conductivity of nanocomposite reaches 110 S/m at t = 50 nm and σ_f_ = 9 × 10^7^ S/m, while at t < 21 nm, the nanocomposite is insulated. It can be observed that the electrical conductivity of PNCs is directly related to the conductivity of nanowires and the thickness of produced interphase. As a result, the higher the conductivity of the nanowires and the thicker the interphase lead to an improvement in the conductivity of nanocomposite. In contrast, the low electrical conductivity of nanowires and thin interphases can insulate the final nanocomposite.

The formation of conductive networks in nanocomposites is facilitated by increasing the interphase thickness around nanowires. The interphase zone around the nanowires is conductive causing the electron transferring. Therefore, electrons can transfer more easily between nanowires, and the electrical conductivity is improved. In addition, according to Eq. (15), a thicker interphase produces a higher effective volume fraction of nanowires, which directly affects the electrical conductivity of nanocomposite. Additionally, since polymer matrices have low electrical conductivity and are generally insulating, it is primarily for the conductivity of nanowires that controls the conductivity of PNCs. The conductivity of PNCs is improved by increasing nanowire conductivity, and the maximum conductivity is attained by adding more conductive nanowires. A major reason for this can be seen in the tremendous differences in the electrical conductivity of polymers and nanofillers^36,57^, which demonstrate the importance of nanowire conductivity to the electrical conductivity of nanocomposite. Therefore, the suggested model indicates that nanowire conductivity largely affects the conductivity of nanocomposites.

**Figure 7:**
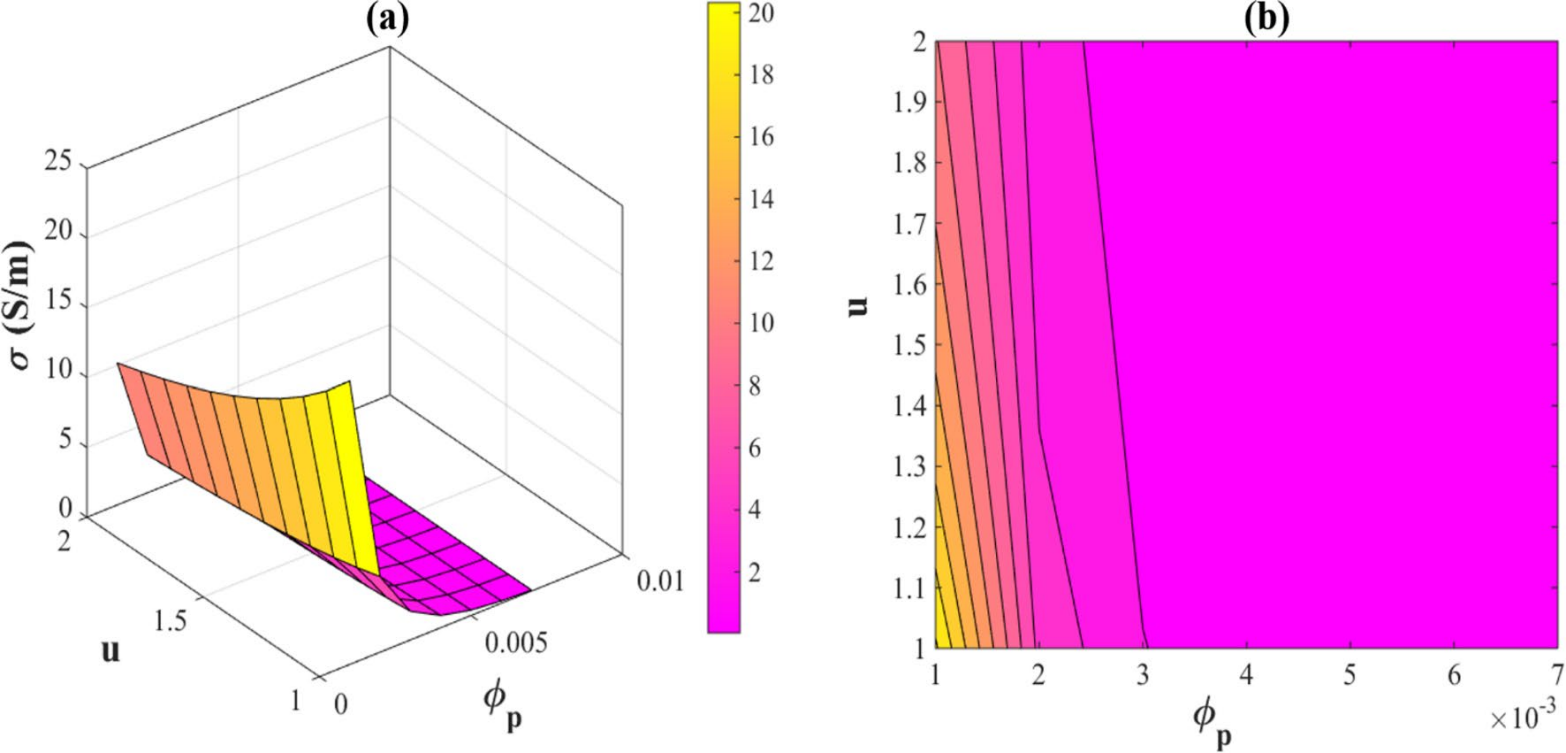
Electrical conductivity of a PNC by *“∅ *_*p*_*” and “u”:* (**a**) 3D and (**b**) contour plots.

Conductivity calculations are shown in Fig. 7 based on *“∅ *_*p*_*” and* “u”. The nanocomposite has a maximum conductivity of 20 S/m at *∅ *_*p*_ = 0.001 and u = 1. Additionally, at *∅ p* > 0.003, the conductivity is 0. Hence, a low percolation threshold and small waviness of nanowires make the nanocomposites more conductive and these parameters differently affect the conductivity.

Percolation volume fraction causes the formation of conductive networks in the systems. The electrical conductivity of the nanocomposite is negatively related to the percolation threshold, since it is well known that a low percolation threshold increases the network percentage (Eq. 17), which increases the electrical conductivity. Literature researches also indicated that a lower percolation threshold causes more conductivity at lower amount of nanoparticles^58^. Therefore, it is reasonable to mention that there is a reverse relationship among conductivity and percolation onset, as offered by the current model. Furthermore, nanowires that are not wavy increase the electrical conductivity of nanocomposites, because a lower range of "u" shows more straight nanowires in PNC increasing the effective length of nanowires. Straight nanowires reach the smallest percolation threshold, which increases the dimensions of networks and conductivity of PNCs^59^. Hence, “u” adversely handles the conductivity of PNCs, as stated by the advanced model.

## Conclusions

An applicable model for the conductivity of AgNW-filled nanocomposites was derived using several key factors including nanowire volume fraction, aspect ratio, percolation threshold, interphase size, tunneling distance, filler waviness and nanowire conductivity. Experimental results confirmed the predictions of the offered model. Furthermore, the effects of dissimilar factors on the electrical conductivity of the nanocomposite were investigated. The mathematical studies indicated that the superficial energies of medium and silver nanowires did not affect the electrical conductivity of PNCs. Therefore, these terms were not taken into account in the proposed model. A minimum conductivity is observed at λ > 3.5 nm or f < 0.37, demonstrating that a lower network percentage and a bigger tunneling distance result in a reduction in the conductivity of nanocomposite. In addition, a maximum electrical conductivity of 305 S/m was shown at ∅_f_ = 0.02 and α > 820, indicating that the aspect ratio and volume fraction of the nanowires directly affect the conductivity. Besides, a low percolation threshold and small curvature of nanowires produce a higher conductivity, but a higher percolation threshold than 0.003 causes an insulated sample. Conclusively, a higher volume fraction of nanowires, longer and more-straight nanowires, thicker interphase, and a smaller tunneling distance will lead to higher improvement in the conductivity of PNCs.

## Data Availability

The data that support the findings of this study are available on request from the corresponding author.
